# Targeted redox and energy cofactor metabolomics in *Clostridium thermocellum* and *Thermoanaerobacterium saccharolyticum*

**DOI:** 10.1186/s13068-017-0960-4

**Published:** 2017-11-30

**Authors:** Kyle Sander, Keiji G. Asano, Deepak Bhandari, Gary J. Van Berkel, Steven D. Brown, Brian Davison, Timothy J. Tschaplinski

**Affiliations:** 1 Department of Chemical and Biomolecular Engineering, University of Tennessee, Knoxville, TN USA; 2 Bredesen Center for Interdisciplinary Graduate Research and Education, University of Tennessee, Knoxville, TN USA; 3 Biosciences Division, Oak Ridge National Laboratory, Oak Ridge, TN USA; 4 Chemical Sciences Division, Oak Ridge National Laboratory, Oak Ridge, TN USA; 5 BioEnergy Sciences Center, Oak Ridge National Laboratory, Oak Ridge, TN USA; 6 Present Address: Centers for Disease Control and Prevention, Atlanta, GA USA; 7 Present Address: LanzaTech, Skokie, IL USA

**Keywords:** *Clostridium thermocellum*, *Thermoanaerobacterium saccharolyticum*, Redox, Adenylate energy charge, Targeted metabolomics

## Abstract

**Background:**

*Clostridium thermocellum* and *Thermoanaerobacterium saccharolyticum* are prominent candidate biocatalysts that, together, can enable the direct biotic conversion of lignocellulosic biomass to ethanol. The imbalance and suboptimal turnover rates of redox cofactors are currently hindering engineering efforts to achieve higher bioproductivity in both organisms. Measuring relevant intracellular cofactor concentrations will help understand redox state of these cofactors and help identify a strategy to overcome these limitations; however, metabolomic determinations of these labile metabolites have historically proved challenging.

**Results:**

Through our validations, we verified the handling and storage stability of these metabolites, and verified extraction matrices and extraction solvent were not suppressing mass spectrometry signals. We recovered adenylate energy charge ratios (a main quality indicator) above 0.82 for all extractions. NADH/NAD+ values of 0.26 and 0.04 for an *adhE*-deficient strain of *C. thermocellum* and its parent, respectively, reflect the expected shift to a more reduced redox potential when a species lacks the ability to re-oxidize NADH by synthesizing ethanol. This method failed to yield reliable results with *C. bescii* and poor-growing strains of *T. saccharolyticum.*

**Conclusions:**

Our validated protocols demonstrate and validate the extraction and analysis of selected redox and energy-related metabolites from two candidate consolidated bioprocessing biocatalysts, *C. thermocellum* and *T. saccharolyticum*. This development and validation highlights the important, but often neglected, need to optimize and validate metabolomic protocols when adapting them to new cell or tissue types.

## Background

*Clostridium thermocellum* is a promising consolidated bioprocessing candidate microorganism capable of enzymatically degrading lignocellulosic biomass and simultaneously converting soluble hydrolyzed sugars to ethanol. Metabolic characterization and engineering efforts have afforded large improvements in overall bioproductivity [1, 2], as well as engineering for heterologous production of isobutanol [3]. *Thermoanaerobacterium saccharolyticum* is a noted anaerobic, thermophilic ethanologen which has also been extensively studied and engineered [4]. While it does not possess the lignocellulolytic capability of *C. thermocellum*, its optimum temperature and pH compliment those of *C. thermocellum* and make it a well-suited co-culture counterpart. These two species of bacteria grown together have successfully produced 38 g/L ethanol in a fermentation initiated with 92 g/L of crystalline cellulose [2].

Previous metabolic engineering efforts toward improving ethanol bioproductivity in *C. thermocellum* and *T. saccharolyticum* have largely focused on carbon forcing [2, 4, 5]. Eliminating competing fermentation end products in these two organisms did not result in maximal ethanol yield on a carbon basis. In *C. thermocellum*, such efforts increased ethanol yield, but failed to decrease carbon flux to other unwanted products, such as amino acids [6], where pathway disruption would likely generate a conditional lethal strain.

The native redox metabolism has been the sole source of reductant enabling ethanol production in these two organisms as yield and overall productivity have improved, and overall conversion and substrate utilization have increased, and larger flux demands have been placed on cellular metabolism. *C. thermocellum* intracellular redox dynamics are unconventional and still being fully elucidated [1, 7–9]. Increasing expression of the genes encoding an Ferredoxin:NAD+ oxidoreducase (*rnf*) in *C. thermocellum* was able to increase ethanol yield by 30% [7], while deleting the genes enconding an NADH-dependent reduced ferredoxin:NADP+ oxidoreductase (*nfnAB*) in *T. saccharolyticum* caused a 30% decrease in ethanol yield [10]. Enabling the bifunctional alcohol dehydrogenases to accept both NADH and NADPH to facilitate ethanol conversion, rather than NADH alone, improved tolerance to ethanol [11], and increased ethanol yield by 37.5 and 73% in *C. thermocellum* and *T. saccharolyticum*, respectively [12]. It was identified through metabolic modeling that *C. thermocellum* does not re-oxidize reduced ferredoxin fast enough to support the fermentative metabolism, leading to metabolic stalling at the pyruvate to acetyl-CoA metabolic node [9], thus highlighting the large effect NADH-dependent ferredoxin re-oxidizing activity of Rnf has on metabolic flux and ethanol productivities. Rate limitation at the catabolic step of acetyl-CoA synthesis from pyruvate is further supported by a metabolomic pulse-chase study that used ^13^C labeled cellobiose to show the unlabeled fraction of pyruvate decreased more slowly than the central glycolytic metabolites upstream of pyruvate [13]. This slower-than-expected depletion of unlabeled pyruvate may also be due to unlabeled CO_2_-derived carbon being assimilated into pyruvate from the reversible activity of Pyruvate–Ferredoxin Oxidoreductase [14], a process which is also redox-driven and can impact ethanol productivity. These in vivo studies suggest that it is intracellular redox state and redox-driven thermodynamic limitations of key metabolic reactions that are now limiting further improvements in yield and overall productivity of ethanol in these microorganisms. A clear and validated assessment of intracellular redox cofactors would help the mechanistic understanding of this limitation further and help identify strategies to increase redox-dependent metabolic flux toward the production of ethanol. Redox-centered metabolic engineering enabled *Yarrowia lipolytica* to produce fatty acid methyl esters at the highest yield and productivity achieved [15]. The performance metrics achieved simultaneously met final titer and productivity objectives (and falling just 4% shy of the yield objective) needed for cost-effective production of Biological Renewable Diesel Blendstock [16]. Similarly, growth of *Pseudomonas putida* in a bioelectrochemical cell in media containing soluble redox mediators allowed it to produce 2-keto-gluconate at 90% of theoretical maximum yield [17].

Different metabolomic techniques used previously to estimate nicotinamide redox cofactors in *C. thermocellum* have given NADH/NAD+ ratios that span a large range [18, 19] and, because of the disparity, offer little metabolic insight beyond intra-experiment relative comparisons. As the relative concentrations of these two metabolites are a tightly regulated parameter [20], it is unlikely *C. thermocellum*, grown and sampled under similar conditions in these studies, is allowing the relative abundance of these metabolites to vary so much. Reliable and validated determination of NADH/NAD+ redox couples will assist in estimating reaction directionality and net flux ratios [21] of critical redox reactions in *C. thermocellum*. Intracellular concentrations, redox state, and adenylate energy charge can make metabolic models more accurate and representative, and elucidate energetic limitations in *C. thermocellum* metabolism. Furthermore, redox cofactor measurements can help understanding of cofactor requirements and interchangeability between charge-carrying species in *C. thermocellum*, and help identify pathways responsible for electron yield losses (in this case defined as electrons that are not being directed toward biomass or ethanol production).

Redox and energy metabolites are known to be chemically labile and susceptible to degradation under routine laboratory handling [22]. We hypothesize that the extraction and detection protocols being used are affecting reported measurements and need optimization and robust validation. Typically, upon adapting metabolomic methods developed for one microorganism for use in other microorganisms, a small number of validation experiments are done addressing a few concerns, but rarely are protocols validated comprehensively. We have identified the many common issues as critically affecting metabolite extractions from microorganisms and biological tissues in general, which sometimes go un-validated before they are adapted and employed.

### Critical aspects of metabolomics methods

#### Adenylate energy charge

Many studies use the adenylate energy charge (AEC) as an intrinsic efficacy indicator of metabolomic extraction and detections. The adenylate energy charge is known to be maintained between 0.80 and 0.95 in most cells [23]. This value is relatively static in growing microorganisms, particularly anaerobic organisms [22]. In facultative anaerobes, the adenylate energy charge only undergoes small and transient changes, upon major shifts in growth state, such as shifting to anaerobic growth from aerobic growth [22]. The AEC is known to be tightly regulated, and is also sensed and responded to by elements of cell state regulation [24, 25]. For these reasons, the AEC is often used as an indicator for overall cell well-being [26, 27], and a decreased AEC can be a proxy for the magnitude of stress induced from experimental treatments [28, 29].

ATP is known to be a particularly labile metabolite [22], as well as the most abundant of the three adenylate nucleotides used to calculate the adenylate energy charge. The ability to observe high and physiologically relevant adenylate charges in metabolomic datasets is a key indicator of adequately careful and reliable metabolite extraction and detection. A low adenylate energy charge may indicate that one or more processing steps could be degrading ATP, as well as other exceedingly labile metabolites. The regulated stability of the AEC, and the ability to detect adenosine cofactors alongside other metabolites, makes the AEC an ideal quality control indicator of metabolomic extractions from actively growing cells.

#### Solvent/extraction and quenching

While rapid and complete metabolic quenching is important to metabolomic extractions, an equal requisite is to quench cell metabolism and extract cellular metabolites in a way that preserves labile metabolites. Other ways to protect labile metabolites are through the introduction of chemical protectants to the extraction protocols, such as redox and pH buffers. Protection of nicotinamide species with the use of chemical additives is specific to cells and tissues, whereby each cell/tissue type requires a specific protocol [30, 31]. Previous reports which quantify nicotinamide and adenosine cofactors show it is possible to preserve these labile species through minimal, cold handling alone, without the need for chemical protectants. The solvent mixture chosen was found to be a superior global metabolomic extraction solvent, developed with special consideration for extracting nucleotides [32]. This solvent mixture is amenable to global metabolomic profiling, and achieves metabolism quenching and extraction simultaneously [33, 34], important to minimizing sample handling.

#### Washing, centrifugation, and metabolite leakage

Washing steps are often included in metabolomic quantification protocols of intracellular metabolites, to remove extracellular species and media components prior to extracting metabolites. Washing cells can cause metabolites to leech from the cells in substantial quantities [35–38]. We are unaware of any precedent to show washing of cells is necessary during fast-filtration metabolomic extractions. We have assessed media supernatant and spent culture supernatant for the metabolites of interest in this study, and found they were not present in either (data not shown). Some studies introduce a correction to metabolite concentrations by first attempting to quantify metabolite leeching, and then using these leakage yield losses to ‘correct’ metabolite quantifications [39, 40]. The amount of metabolite leakage may change as a function of experimental condition, cell growth state, as well as other parameters that are often experiment specific, necessitating careful quantification of leakage losses for each experiment. If the amount of leakage is large and variable, these yield corrections may not viably represent leakage across experiments and replicates.

Centrifugation steps can last on the order of ~ minutes. If centrifugation is done before metabolic quenching, the metabolite profile can change, even at decreased temperatures [41]. If centrifugation is done after metabolism quenching, metabolite degradation or metabolite leeching from cells may ensue.

With sub-second turnover rate of many reactions and degradation mechanisms involving these metabolites, quenching within the timescales of these reactions is preferred, as is offered by direct cooled solvent quenching. Studies have shown metabolites of upper glycolysis to have turnover rates of < 1 s, even at 0 °C [42–44], though most rapid quenching/extraction methods involve submerging cells + media directly into cooled extraction solvent, which can result in > 20% leakage of some metabolites [45, 46].

#### Metabolite mass spectrometry signal suppression

The suppression of mass spectrometry signals of metabolites is often encountered in metabolomic protocols that do not separate cells from their spent media prior to metabolite extraction [47, 48]. The IDMS (isotope-dilution mass spectrometry) method, or one of the many derivatives of this method [49], is used to check for and correct signal suppression in metabolomics. Labeled extracts used in IDMS themselves are subject to degradation from handling and storage. Metabolite degradation in IDMS standards which are spiked into sample extracts could incorrectly skew correction factors and lead to inaccurately corrected data. Labeled metabolites, particularly for global metabolomics, are typically produced by growing *Escherichia coli* on 100% labeled carbon substrate in minimal media, extracting those metabolites and spiking this extract into samples to be analyzed. This method of preparing labeled metabolites for IDMS results in metabolite pools that are incompletely labeled [32], requiring additional data corrections. This method introduces extensive data augmentation, an additional potential source of error. We are not aware of a published incidence of signal suppression in metabolomic studies employing fast-filtering and solvent extraction for the targeted subset of metabolites assayed for in this study. An alternative to IDMS, particularly amenable to targeted metabolomics of a small number of metabolites, is to validate that there is not ion suppression of target metabolites occurring prior to analyzing experimental samples. It is important to re-affirm this upon introducing new or different experimental or sampling conditions.

### Other nicotinamide metabolite quantification methods

#### Native in vivo fluorescence and fluorescent biosensor detection of pyrimidine nucleotides

Nicotinamide cofactors natively fluoresce and this fluorescence can be used to quantify them in vivo. The emission spectra for NADH and NADPH are similar (abs. 366 nm, emit. 460 nm) [50] and instantaneous fluorescent measurements cannot discriminate between the two species, nor can they discriminate between bound and free forms of these cofactors. The standard potential of NAD(H) and NADP(H) differs slightly [51], as does the intracellular concentration and often the relative ratios of the oxidized and reduced species and enzymes typically do not use both interchangeably. As such, these two charge carriers are not equivalent within the cell, and combined measurements of both, as reported from chemical autofluorescence, are inappropriate when attempting to infer redox information about one or the other. Further complicating in vivo analysis of native forms is the fact that other biomolecules can interfere with fluorescent measurements, such as FAD and other flavins [52, 53]. The fluorescence decay properties of pyrimidine cofactors are different from each other and thus allow for their determination individually in vivo [54–56]. Coupling fluorescence decay analysis and spectral decoupling methods allows for the in vivo differentiation of free and protein-bound NADH [56, 57]. Fluorescence lifetime techniques require specialized equipment, cell preparations, and techniques which would likely result in cells being in a state not representative of growth. These techniques are also not amenable to large numbers of samples and replicates, nor are they compatible with simultaneous determination of other metabolites through methods such as global metabolomic profiling.

Toward achieving NADH/NAD+ determinations during active growth states and increasing throughput and flexibility of analysis, abiotic and protein-based biosensors have been developed to assay the in vivo redox potential of NADH/NAD+. Biotic biosensors have been developed to measure NADH/NAD+ redox state directly, largely leveraging the differential affinity of the Rex transcription factor for NAD+ and NADH [58–60], or indirectly, through the use of coupled reporter systems [61]. While these biological redox sensor systems can give measurements under a variety of growth states, they are vulnerable to interference from pH, other nucleotides/metabolites, temperature [62], and the exogenous redox potential [63]. Their use to quantitatively measure NADH/NAD+ requires careful control and calibration of many parameters which affect their performance [62] and, given the difficulties in calibrating and standardizing these biosensors for all possible conditions and interferences, measurements derived from these biosensors are usually reported as relative and differential after being normalized to an appropriate control. Furthermore, genetic biosensors must be genetically integrated and functionally validated for each adapted use, a particular challenge to metabolic investigations of non-model organisms whose heterologous genetic expression tools are still being developed [64]. Although of relevance to *C. thermocellum* and other biotechnologically relevant thermophiles, there is a class of NADH/NAD+ biosensors based on T-Rex, the Rex protein from *Thermus aquaticus* [65], and a thermophile with an optimum growth temperature of 70 °C; however, the biosensor itself has not been applied, tested, or adapted at elevated temperatures.

Abiotic biosensors based on activated surface chemistries synthesized specifically to record amperometric responses to oxidation of NADH extracted from cells. These devices assay NADH from biological extractions, which must be extracted/prepared, wherein doing so requires the same considerations as addressed when preparing extracts for LC–MS/MS. As with biotic biosensors, these devices are susceptible to interference from other biomolecules present in extracted matrices. Unlike biological in situ biosensors, assay conditions can be carefully controlled, allowing for calibration and absolute quantification of NADH. The detection limit for NADH in these devices is similar to those reported for MS/MS methods and in vivo fluorescent methods (~ 20–160 nM) [66, 67].

#### Enzymatic cycling assay

Enzyme cycling assays are also commonly used to detect NADH and NAD+ [30, 31]. Extractions using ~ 1 M acid or base (depending on the metabolite being assayed for) are commonly employed with these assays. This protocol is able to chemically stabilize and detect picomolar concentrations [30], which is well below the concentrations typically found in metabolite extractions. Extensive tissue specific requirements are typically required to preserve NADH and NAD+ from degradative ability of extraction matrices [30, 31]. Extraction involving high concentrations of acid or base is destructive and not amenable to concomitant measurements of other metabolites. Aside from assessing recovery in ‘blank’ or matrix-laden extractions, there are few other options to assess metabolomic data quality with this method. This assay is not amenable to detecting NADH and NAD+ metabolites extracted in organic containing solvents and co-extraction of adenylate cofactors to determine the AEC is not possible.

In conducting cycling assays, unwanted nicotinamide species (NADH, NAD+) in each extraction are degraded away prior to quantifying the corresponding other species. While this was shown to occur to completion in pure solution [41], many cycling assay development and adaptation papers mention incomplete destruction of unwanted nicotinamides in the extractions, which then can interfere with the assay. Incomplete destruction and conversion of interfering species is difficult to detect and account for, even when assaying for recovery of exogenously added metabolites. When assaying for low quantities of metabolites, these interferences can have a large effect. Not only does fast filtering utilize quenching and extractions designed to preserve the native state of all metabolites extracted, but all metabolites are also analyzed for simultaneously, rather than separately from different extractions, eliminating the possibility of overestimating the concentration of nicotinamide metabolites or the entire nicotinamide pool.

#### In vivo NMR

In vivo NMR has been used to detect intracellular metabolite concentrations in various microbes [68], including redox and energy cofactors [69]. In vivo determination does not require metabolites to be extracted from cell biomass prior to detection and quantification. The main drawback from NMR metabolomics is the relatively low detection limit, which is often many orders of magnitude above metabolite concentrations found in metabolomic extracts [70]. In vivo NMR metabolomic methods offset this limitation by detecting metabolites from highly concentrated material, in situ or as extracts from large amounts of cell biomass. In vivo intracellular adenylate cofactor determination of *C. thermocellum* [71, 72] used highly concentrated cells and, though the cells are metabolically active, the metabolic state of these cells may not represent the metabolic state of actively growing and fermenting cells. Metabolic or metabolomic inferences between the two cell states may be only tangential. Ex vivo NMR-based metabolomics circumvent low detection limit limitation by extracting metabolites from relatively large amounts of cell biomass [69].

In this study, we conduct a series of experiments toward qualifying a protocol for the reliable simultaneous determination of NAD(H), NADP(H), and A(T,D,M)P. Toward adopting and optimizing a protocol originally developed for use with *E. coli* [32], we obtain intracellular energy and redox cofactor concentration measurements, as well as validation experiments which address common metabolomic concerns that introduce large artifacts in other metabolomic extraction and detection protocols: metabolite leakage, degradation, yield losses, and mass spectrometer signal suppression. We use a solvent quenching/extraction of filtered cell biomass followed by direct determination of metabolites using LC–MS/MS modified to include a minimum number of processing steps, and occurring at or below 0 °C in an anaerobic environment. We have omitted centrifugation and washing steps to avoid metabolite leakage and, because we observe no matrix-induced mass spectrometry signal suppression, omit any signal correction methods (e.g., isotope-dilution mass spectrometry, standard additions) as well. These validations also bound the quantitative possibilities of our results and add confidence to the measurements. Similar validations might be used when adapting metabolomic methods to other cell or tissue types.

## Results

### The adenylate energy charge and metabolomic protocol efficacy

A key metric and indicator of metabolite extraction efficacy and quality typically referenced is the adenylate energy charge (AEC) recovered from observed metabolites, when possible. We followed this metric while adapting the protocol of [32] and augmenting it for use with *C. thermocellum* and *T. saccharolyticum*. Through our adaptation of this protocol, we aimed to alter and improve our protocols to attain and observe increasingly higher AEC values in metabolite extracts. We find the ability to preserve ATP generally indicates we were preserving other labile metabolites as well, such as NADH (Table 1.)

**Table 1:** Adenylate energy charge (AEC) improvements observed through protocol development

Date	AEC	NADH (µM)	ATP (µM)	Protocol improvements from previous
1	0.411 ± 0.017	0	0.246 ± 0.031	Ethanol-based solvent, aggressive sonication protocol, extraction temperatures reached ~ 50 °C
2	0.804 ± 0.009	0.123 ± 0.006	2.31 ± 0.13	Fast-filtering extraction and aqueous/organic extraction solvent, adapted from [32] with modifications
3	0.91 ± 0.01	1.17 ± 0.06	3.61 ± 0.02	Further improved handling, removed formic acid from extraction solvent

Metabolite extraction efficacy increases with the AEC ratio and the AEC ratio is an appropriate quality control metric to use, if possible, when extracting metabolites. Through making changes that increased the AEC, we concomitantly saw that we could extract and preserve higher concentrations of the two most labile metabolites targeted in this study, ATP and NADH.

### Sufficient metabolite recovery through a single extraction

**Fig. 1:**
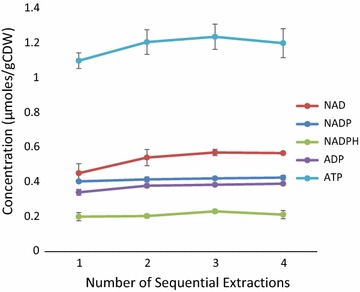
Cell biomass was extracted multiple times to determine if extracting biomass multiple times is necessary to recover all metabolites present in collected biomass. Using this protocol, extracting cell biomass once is sufficient for complete extraction and quantitation of metabolites. AMP and NADH were unable to be detected in this experiment

Often in metabolomic studies, sample biomass is extracted multiple times [47, 73], presumably as a precautionary measure to ensure complete extraction. We aimed to determine if extracting biomass multiple times is necessary to extract redox and energy metabolites of interest from *C. thermocellum* and *T. saccharolyticum*. We extracted unwashed cell biomass entrained on a nylon filter into 2 mL of chilled extraction solvent. To extract cell biomass more than once, the cell-containing filter was washed with an additional 1 mL of fresh solvent, to prevent carryover, and then transferred to a fresh chilled 2 mL of extraction solvent. Within error, a single extraction of cell biomass is sufficient to extract metabolites from *C. thermocellum* using the protocol developed herein (Fig. 1). Furthermore, the sample-to-solvent ratio is sufficient for metabolites to be extracted in a single extraction.

In doing this experiment, we were unable to detect all seven metabolites we were attempting to detect. To prepare extracts for LC–MS/MS analysis, extract from each of the four sequential extractions were combined as shown in Fig. 6, so as to make detection of incremental increases in subsequent extractions possible. In doing so, the extract concentration of all metabolites was ~ 1/4 of concentrations typically observed for *C. thermocellum*. Although this finding and suggestion is made on the basis of detecting five of the seven targeted metabolites in this experiment, the levels of corresponding cofactor pool counterpart metabolites which were detected are not varying in extracts of biomass extracted multiple times. As such, it is not likely that multiple extractions are necessary or will enable better metabolite recovery and detection.

### Recovery losses through handling in fast filtering and collection

Through adding exogenous metabolites into the extraction solvent, and performing a ‘mock’ extraction (see “Methods”) using a filter with no cell biomass entrained in it, we enumerated metabolite recovery losses which occur during a metabolite extraction. We assayed for yield losses using metabolite concentrations typical of those found in metabolite extracts of *C. thermocellum* and *T. saccharolyticum*.

**Fig. 2:**
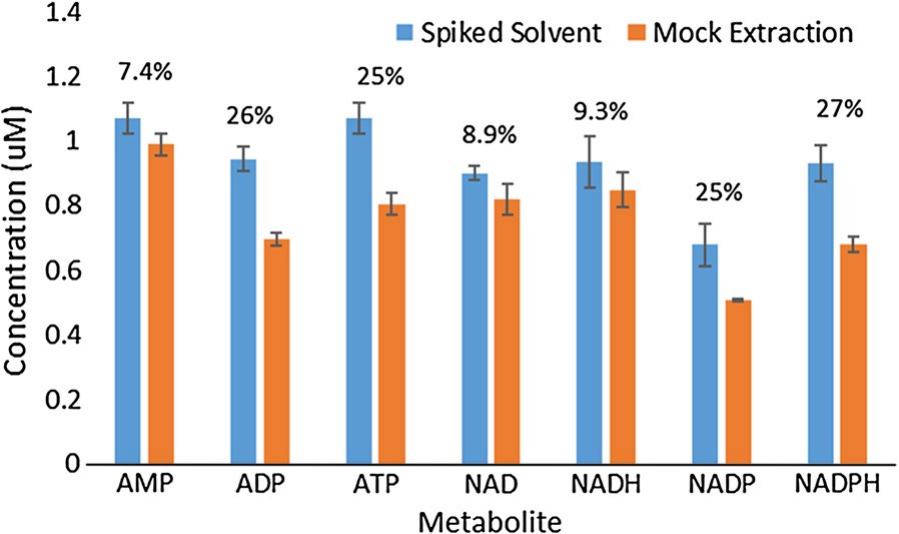
Extraction solvent containing exogenously added metabolites was used to conduct a ‘mock extraction’ to assess metabolite losses due to handling. Blue bars indicate amount of metabolite quantified in solvent containing spiked metabolite. Orange bars indicate amount of solvent quantified in spiked solvent after one pass through a mock extraction. Noted above each metabolite is the percentage of each metabolite lost during mock extractions relative to the amount present in the spiked solvent. A(T,D,M)P and NAD(P)(H) are susceptible to handling-related losses

ATP, ADP, NADPH, and NADP incurred the largest recovery losses (Fig. 2). Less than 10% of NADH and NAD+ were lost during sample handling. NADH and NAD+ loss differences are among the smallest of the seven redox and energy metabolites targeted in this study. The yield losses quantified here cannot account for large differences in NADH/NAD+ ratios observed in this study and others quantifying this parameter in *C. thermocellum* [18, 19]. Reduced nicotinamide cofactor losses cannot be accounted for in their oxidized counterparts and ATP does not appear to be hydrolyzing to ADP and AMP. Yield losses due to handling were observed in all metabolites, to varying degrees. We observed one of the largest recovery losses in ATP, though are still able to observe relatively high and physiologically relevant AEC ratios. Yield decreases across all seven metabolites suggest that metabolites may have been lost to sorption to a surface or material contacted during the extraction protocol. One reason glass materials were chosen was to minimize such losses. The most likely source for these sorptive losses is the nylon filter. It may be prudent to further assess different filter material for their sorption properties and select filter media displaying appropriately low metabolite adsorption. As this mock extraction was carried out using filters containing no cell biomass, sorptive properties of filters may be different when they contain cell biomass. While these losses are non-trivial, it will serve as a basis and starting point for estimating true intracellular metabolite concentrations and inform future protocol improvements aimed at reducing these losses.

### Storage stability at − 80 °C

**Fig. 3:**
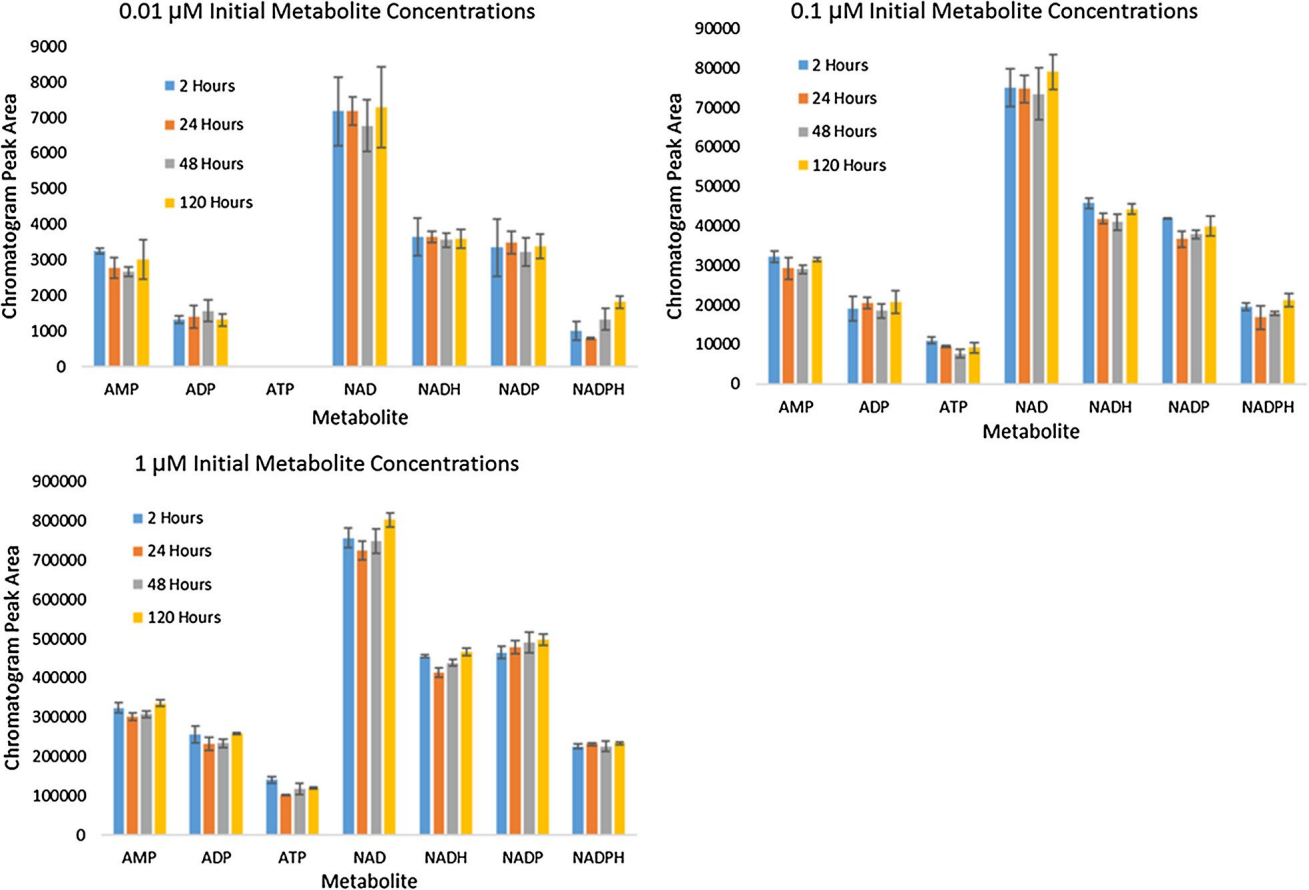
Storage stability of metabolites was assessed over 5 days at − 80 °C in extraction solvent at concentrations 0.01–1 µM. All metabolites appear stable under these storage conditions

These labile metabolites do undergo degradation at sub-zero temperatures [41]. To validate a typical storage protocol, an equimolar mixture of exogenous metabolites was prepared in fresh extraction solvent. Aliquots were frozen for prescribed lengths of time and metabolite concentration was analyzed for stability over time. Each sample mixture was frozen and thawed once. Figure 3 shows metabolite concentrations of these seven metabolites, added exogenously, after being stored at − 80 °C for various lengths of time. All metabolites were stable when frozen at − 80 °C for up to 5 days.

### No matrix-induced signal suppression of targeted metabolites

Signal suppression of the metabolites was assayed by first injecting either *C. thermocellum* cell extract or extraction solvent. A mixture of the seven metabolites of interest (0.25 µM each metabolite) was introduced into the stream of column eluent, creating a steady-state mass spectrometry signal for each metabolite. The resulting combined signal was monitored for signal decrease at the expected retention time and *m/z* value corresponding to each metabolite. Small signal increases seen at the expected retention times of some trials appear as the result of metabolites present in the initial injected sample. Any signal decrease at the expected retention time and *m/z* value would indicate suppression of the metabolite signal by the cell extract matrix.

**Fig. 4:**
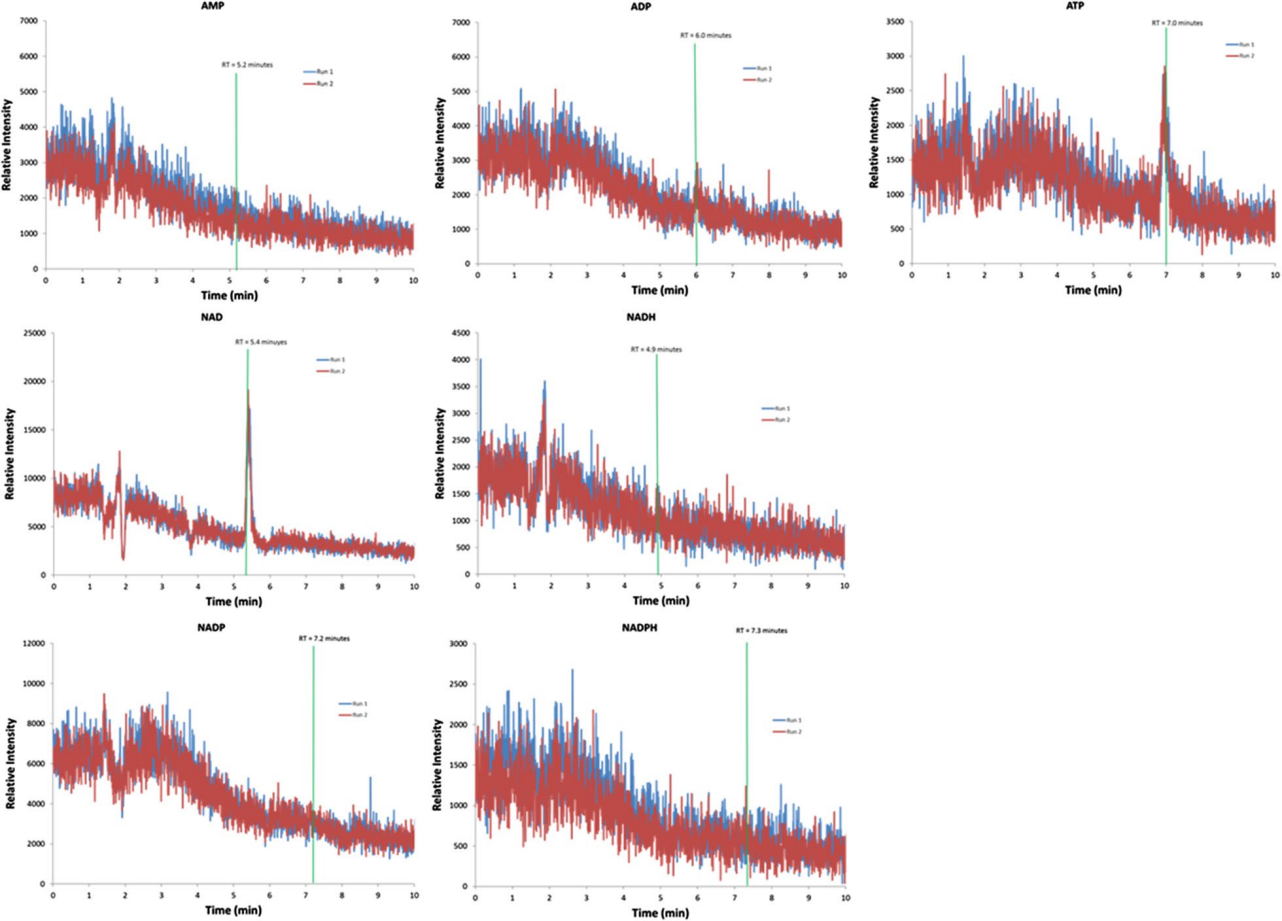
Mass spectrometry signal suppression brought about by cell extract components were assessed as deflections in steady-state metabolite signals (created by infusing a mixture of the seven metabolites of interest in this study into the chromatography column eluent). Predetermined retention times for each metabolite (indicated by green bars) were monitored for signal deflection, which would indicate signal suppression by the cellular extract matrix. No signal suppression was observed from extraction solvent or extraction matrix at expected retention times for metabolites. Each steady-state metabolite signal was assayed for signal suppression in the presence of cell extract twice. Both assays are shown overlaid (red and blue lines)

No signal decreases were seen at any of the expected retention times at any of the *m/z* signals in the presence of either *C. thermocellum* cell extract or extraction solvent, indicating that neither interferes with detected signals assayed for in this study (Fig. 4). Instances of signal suppression were observed, but were found outside of the expected retention time, such as at ~ 2 min at *m/z* corresponding to NAD+. As cell extract matrix resulting from this extraction protocol does not produce any mass spectrometry signal interference for these seven metabolites, there is no need to correct for signal suppression.

### Nicotinamide and adenylate cofactor extractions from *C. thermocellum* and *T. saccharolyticum*

We have only validated other extraction aspects for *C. thermocellum;* however, we report intracellular metabolite concentrations for both *C. thermocellum* and *T. saccharolyticum* (Table 2). We have extracted and detected these redox and energy metabolites from a strain of *C. thermocellum* in which the bifunctional *adhE* gene has been removed [74], as well as its genetic parent strain. We find the Δ*adhE* strain to have a larger NADH/NAD+, as is expected without the function of the NADH-dependent enzyme. This relative difference in the NADH/NAD+ ratio has been shown before, though the magnitude of the ratios was much higher [19]. We also observe a much lower NADH/NAD+ ratio in *T. saccharolyticum* than was observed previously.

**Table 2:** Varying adenylate charge ratios observed across species highlights the need to develop protocols specific for each species/strain

Species/strain	Genotype	Fermentation capabilities	Metabolite concentrations (μmol/g CDW)							NADH/NAD+	NADPH/NADP+	Adenylate energy charge
			AMP	ADP	ATP	NAD	NADH	NADP	NADPH			
*Clostridium thermocellum*												
LL345	Δhpt	Wildtype	0.12 ± 0.047	0.64 ± 0.23	4.22 ± 1.1	1.26 ± 0.4	0.05 ± 0.01	0.15 ± 0.03	0.17 ± 0.07	0.04	1.13	0.91
LL1111	Δhpt ΔadhE	< 5% of wt ethanol	0.53 ± 0.14	1.87 ± 0.54	5.64 ± 1.14	1.83 ± 0.42	0.48 ± 0.23	0.49 ± 0.04	0.71 ± 0.15	0.26	1.45	0.82
*Thermoanaerobacterium saccharolyticum*												
LL1025	Wildtype	Wildtype	0.16 ± 0.078	0.88 ± 0.21	4.4 ± 1.26	2.41 ± 0.51	0.06 ± 0.01	0.52 ± 0.1	0.09 ± 0.02	0.02	0.17	0.89

We also attempted to extract and detect metabolites from a Δ*adhE* strain of *T. saccharolyticum*, though the observed adenylate charge ratio was 0.69, a value too low for reliable metabolomic determination. This was likely due to the incompatibility between this poor-growing strain [19] and this metabolomics protocol. As the strain grew poorly and unpredictably, it did not display a clear log phase of growth and it was difficult to discern the growth state of the cells. Other studies have circumvented troubles brought about by this severe phenotype by adding yeast extract to growth media. We opted not to do this as previously, yeast extract-containing media had given interfering MS signals when assayed for previously (data not shown).

Contrary to previous findings [19], we find *T. saccharolyticum* to have a lower intracellular NADH/NAD+ ratio than *C. thermocellum*, and a much lower NADPH/NADP+ ratio. In agreement with these previous findings, we find *C. thermocellum* to have a much higher NADPH/NADP+ ratio than *T. saccharolyticum*. *T. saccharolyticum* is a noted natural ethanologen [4] and grows at a much lower optimum pH than *C. thermocellum*, suggesting it may employ far different membrane potential dynamics than *C. thermocellum*. *C. thermocellum* suffers from a large ‘titer gap’ [75], where it is tolerant to a far higher concentration of ethanol than it produces.

We also attempted to apply this protocol to extract and detect these metabolites from the lignocellulolytic thermophile *Caldicellulosiruptor bescii.* In our attempts, the AEC observed in metabolite extracts were 0.6–0.7, below acceptable values. Although our protocol was very similar (with adjustments made to filter an equivalent amount of cell biomass), we obtained very different results, further highlighting the need to optimize and validate metabolomic protocols for each cell type.

## Discussion

The reliable metabolomic determination of labile metabolite detection requires careful considerations, beyond the considerations required of more stable metabolites. We have developed a protocol for extracting and detecting a subset of labile redox and energy metabolites, namely ATP, ADP, AMP, NADH, NAD+, NADPH, and NADP from *C. thermocellum* and *T. saccharolyticum*. Throughout the development of this protocol, we achieved more reliable and higher quality metabolite extractions through minimizing the processing steps of our quenching and extractions, as well as ensuring cold, anaerobic culture handling up until the time samples were diluted and prepared for HPLC separation. Included in this study are a series of validations, meant to assess how various process steps can impact metabolite extraction yield at each process step. Data from these experiments can assess extraction efficacy, inform efforts to further improve this extraction protocol, or provide a format for adapting and optimizing this protocol for use in other species or cell types.

### Adenylate energy charge and reliable quantifications

A hallmark of high-quality metabolomic extractions is the ability to observe high and physiologically relevant adenylate energy charge ratios in metabolite extracts [32, 47]. The AEC is often cited, though briefly, as a sign that metabolites are being preserved in their physiological state [33, 47]. We used this ratio as the main indicator of quality, along with metabolite concentrations extracted and results of the various validations we did, to assess reliability of the targeted metabolomic protocol we have adapted for use in *C. thermocellum* and *T. saccharolyticum*. Based on relative increases in extraction yield, and instances of co-degradation (Table 1), we show that the ability to preserve ATP (and observe a high adenylate energy charge) is an indicator that our extraction and detection protocol preserves other labile metabolites as well. While some studies indicate the need for acidic species, such as formic acid [33], to be present in the extraction solvent to reliably extract ATP, we found higher and more consistent AEC ratios in *C. thermocellum* and *T. saccharolyticum* using extraction solvents without formic acid. The addition of formic acid was originally empirically determined to increase adenylate detection when extracted from *E. coli* [76], and was also suggested to aid in denaturing proteins [33], though no data were presented in support of this suggestion. The reason for our empirical finding, that extractions are more effective when formic acid is omitted from the extraction solvent, differs from those made previously may be due to cell-wall structure differences between *E. coli* (gram-negative) and the organisms studied herein (both gram-positive). Gram-negative cell walls are generally considered more impervious than gram-positive cell walls, making these organisms generally more resistant to antibiotics and able to support a chemically isolated periplasmic space. Gram-positive cell membranes, while more structurally resistant to disruption, are typically more porous and susceptible to dyes and detergents. While these cell-wall descriptions are generalities and do not always hold true, e.g., *C. thermocellum* can appear gram-negative when subjected to a gram stain [77], formic acid may not be necessary to extract these metabolites from the cellular matrix largely made up of gram-positive cell walls. Another important observation is that formic acid improved extraction of metabolites form cells grown aerobically, either in liquid culture [76] or grown on filter membranes supported on agar media [32]. *C. thermocellum* and *T. saccharolyticum* are both strict anaerobes. Another study employing a similar extraction protocol to measure intracellular metabolites from a strict anaerobe (*C. acetobutylicum*) also mentions using the same extraction solvent used in this study (40%/40%/20% acetonitrile/methanol/water) without the addition of formic acid [34], suggesting that growing the cells anaerobically or aerobically may determine metabolite extraction efficacy when formic acid is present in the extraction solvent.

### Fast filtering with organic/aqueous solvent precludes matrix-induced ion suppression and simplifies sample handling and analysis

We observe 9–27% yield loss during extraction handling steps of our seven targeted metabolites, with the highest losses coming from NADPH and NADP. Another study mentions not being able to extract NADPH in cold methanol, and achieved higher concentrations with perchloric acid [78], suggesting the use of organic solvents to extract NADP(H) may be suboptimal. Under analogous conditions (exogenously added cofactors, no extract matrix present), 14% of NAD+ and up to 17% of NADH were not recovered when using acid/base extraction and an enzymatic cycling assay [79]. They also mentioned observing higher yield losses of these metabolites when they were present at lower concentrations, and virtually no loss at higher concentrations, emphasizing the need to assay recovery using additions at metabolite concentrations expected in cell extracts. By comparison, we observed recovery losses of NADH and NAD+ of less than 10%. In another study, > 95% of nicotinamides were recovered when coextracting exogenously added chemicals alongside *C. thermocellum* cell biomass [19], though it is unclear what concentration of exogenously added chemical was used and if the concentration used is representative of cell extract concentrations.

The present method is both sufficiently sensitive to detect ~ 10 nM quantities of metabolites in extracts, and is not chemically destructive. Not only does this protocol allow AEC monitoring, but also makes the protocol amenable to development of detection protocols of more metabolites that are likely present in the extract [33]. Furthermore, there is no need for IDMS-based signal correction, as we found no evidence the mass spectrometry signal was being suppressed at the retention times for these seven metabolites. A similar method was used to assess intracellular metabolites in *C. acetobutylicum* [34], and appears to also have been adapted from the same protocol originally developed for *E. coli* [32]. They observed very high AEC values in log phase growing cells, though did not report any other validation experiments such as yield losses incurred at each process step.

To enable more quantitative metabolite determinations, which are imperative for making reliable thermodynamic inferences, accounting for the different types of yield loss can inform protocol improvement. Examples are losses due to leeching (or leakage), degradation, or sorptive losses, and losses during storage, and suppressed signals in detection. This is likely not an exhaustive list of yield losses, but accounts for the most documented sources of metabolite losses.

This fast-filtering protocol does not induce signal suppression during mass spectrometry detection. This is preferable as it does not require extensive sample alterations and data corrections, which both could be potential sources of error in measurements and add processing steps which might reduce extraction and detection reliability.

### Redox dynamics of ethanol-producing anaerobic thermophiles

To assess the performance and sensitivity of this protocol, we extracted metabolites from *C. thermocellum* Δ*adhE* strain (LL1111) as well as its parent (LL375). We also extracted metabolites from *T. saccharolyticum* Δ*adhE* strain (LL1076) as well as its parent (LL1025), though the AEC ratios obtained for the LL1076 strain were too low (0.69) to be considered reliable. We also tested this protocol with strains of *C. bescii*, though AEC ratios obtained for all strains were low (0.60–0.70), and are not discussed herein.

Two other studies [18, 19] have reported values for these metabolites in *C. thermocellum*, and have used protocols much different than the protocol developed and used in this study. We observe large differences in the NADH/NAD+ and NADPH/NADP+ ratios between all three studies, while all three studies reported similar intracellular concentrations for these metabolites. This suggests that the values obtained and reported are heavily influenced by the extraction and detection protocol used [18] extracted metabolites from wildtype *C. thermocellum* as well as two strains that had been exposed/adapted to 3 g/L ethanol. Only in [18] was it possible to assess the AEC, as [19] used an extraction protocol which does not preserve adenylate cofactors. Nicotinamide cofactors were recently reported from a suite of wildtype and engineered strains of *C. thermocellum* and *T. saccharolyticum* collected using a different extraction and analysis method [19]. Three of the strains analyzed were also assessed in the present study.

All but one of the AEC ratios calculated from reported values in [18] are below the physiological range of 0.8–0.95 for actively growing cells [23]. The WT and EA0 samples had observed adenylate charge ratios of 0.737 and 0.699, respectively, while the EA3 sample had an AEC of 0.873. The low AEC observed were likely due to the extraction protocol used; a multistep quenching and extraction followed by high pressure cell cracking, centrifugation, and filtering, all potential sources of degradation or extraction yield loss. We observed approximately the same concentration of adenylate and nicotinamide cofactors as was observed in this study using a quenching and extraction protocol with far fewer steps, which can be completed in much less time overall and in which cell metabolism is quenched within a few seconds rather ~ 10 min. Cui et al. [18] also grew *C. thermocellum* in a media containing 6 g/L yeast extract [80], which may have both altered metabolite states and, as we found in our work, may have been a source of MS signal suppression, justifying their use of stable isotope dilutions to correct for any signal suppression. Between this study and two other studies mentioned, reported values of NADH/NAD+ in wildtype, unperturbed, *C. thermocellum* strains range from 0.04 to 0.48. NADPH/NADP+ values range from 0.41 to 2.1. Redox couple ratios for the nicotinamides are reported as being much more reduced in the studies of [18, 19] than this study. Between these three studies, intracellular nicotinamide cofactor concentrations range from between 0.05 and 1.64, spanning two orders of magnitude. For comparison and context, NADH/NAD+ ratios change only twofold when *C. acetobutylicum* shifts its metabolism from acidogenic to solventogenic [34], a major metabolic change as indicated by large shifts in intracellular metabolite profiles and AEC.

*Clostridium thermocellum* uses a bifunctional alcohol dehydrogenase to produce most of the ethanol it produces [74]. This reaction is NADH dependent and the amount of ethanol produced changes in response to environmental and genetic changes [1, 81], suggesting that a major determinant for ethanol production is the state of the NADH/NAD+ redox couple. As the Δ*adhE* strain does not have this enzymatic capability, relatively higher NADH/NAD+ ratios are expected in this strain, as observed in both this study and in [19].

### Higher NADH/NAD+ in *C. thermocellum* than *T. saccharolyticum*

The NADH/NAD+ ratios observed in this study were approximately an order of magnitude smaller than those observed by [19]. The reason for this difference remains unclear. As many biochemical reactions can exist in a state near equilibrium, it is important to correctly determine the NADH/NAD+ ratio when making thermodynamic or directionality inferences, and a possible range spanning an order of magnitude for the same species grown in the same media and in similar conditions does not lend confidence to such inferences.

We observe a slightly higher NADH/NAD+ ratio in *C. thermocellum* when compared to mid-log NADH/NAD+ ratio of *T. saccharolyticum*, though we observe relatively low intracellular NADH concentrations in both species. Higher NADH/NAD+ ratios were also observed previously in *T. saccharolyticum* relative to *C. thermocellum [* 19 *]*. A strong inverse relationship was observed between intracellular NADH/NAD+ and GAPDH activity in *C. acetobutylicum*, particularly in the range of NADH/NAD+ of 0–0.2 [82]. Another study demonstrates a direct link between GAPDH activity and flux through lower glycolysis in *Lactococcus lactis* [83]. This enzyme is part of the central glycolytic ‘thermodynamic bottleneck’ [21], a set of reactions with noticeably small free energy changes and operating at relatively low net flux ratios. This enzyme is likely operating very near equilibrium and relatively reduced GAP dehydrogenase activity, resulting from the observed higher NADH/NAD+, may contribute ultimately to the disparity in ethanol productivity between *C. thermocellum* and *T. saccharolyticum*. NADH/NAD+ ratio is also driving reactions elsewhere in glycolysis of *C. thermocellum*, namely the Pyruvate:Ferredoxin Oxidoreductase (PFOR) in *C. thermocellum.* This enzyme has shown ‘reverse’ flux in *C. thermocellum*, fixing CO_2_ and synthesizing formate during fermentative growth [14]. If the reaction catalyzed by PFOR is operating opposite to glycolytic flux, and has a relatively low Δ_*r*_ *G* ^o^’ of − 20 kcal/mol [84], it is reasonable that, given amenable concentrations, other glycolytic reactions might be operating similarly, at relatively low net flux ratios. In this way, the NADH/NAD+ redox couple would be heavily influencing the direction of flux for this reaction and potentially other reactions in which it participates.

### Free vs. bound cofactors

It is only free redox cofactors that contribute to the reaction potential of those reactions which they participate in, and it is this potential we desire to estimate with quantitative or near-quantitative estimations of metabolite concentrations. It is suggested that acetonitrile in extraction solvents sufficiently denatures all proteins, thereby releasing would-be bound cofactors and making them available for extraction [85], though it is unclear whether this solvent mix is capable of releasing all enzyme bound cofactors [32]. It has been reported that a substantial portion of the nicotinamide cofactor pool is protein bound in mitochondria [57], while [33] found in *E. coli* that, globally, metabolites were largely in the free, unbound state within the cytosol. The whole-cell total NAD+/NADH ratio and the free/unbound NAD+/NADH portion of the pool differed by an order of magnitude in *Saccharomyces cerevisiae* and the thermodynamic potential of each of these pools would be interpreted differently [86], though total NADH/NAD+ and free NADH/NAD+ were found to approximate each other in human erythrocytes in a variety of growth states [87]. Sun et al. [88] used cytosolic lactate/pyruvate ratio as a proxy indicator of the free cytosolic NADH/NAD ratio, though the lactate/pyruvate ratio itself was labile. Fluorescent lifetime imaging and quantification measurements (FLIM) can also discern free from bound NAD(H) and, thus, the free cytosolic NADH/NAD+ ratio [54], though the growth state of cells prepared for this technique may not approximate that of actively growing cells. It is unclear how well these estimates approximate those of the free, unbound portion of the pool and, thus, the thermodynamic driving force of these unbound pools.

## Conclusions

To enable determining intracellular NAD(H), NADP(H), ATP, ADP, and AMP concentrations in *C. thermocellum* and *T. saccharolyticum*, we have adapted and validated a cold solvent, fast-filtering protocol adapted based on a protocol developed for use with *E. coli* [32]. This protocol is validated on the basis of metabolite recovery, storage and handling stability, mass spectrometry signal suppression, and the ability to recover physiologically relevant adenylate energy charge ratios in extractions. We compare our results with those of two similar studies utilizing different determination methods to quantify these metabolites in *C. thermocellum* and *T. saccharolyticum*. We find that our protocol recovers high adenylate energy charges and physiologically meaningful values for NADH/NAD+ and NADPH/NADP+ that are validated by other metabolomic data in the related literature. Due to tissue and extraction matrix specific needs, such validations can and should be used when adapting this and other metabolomic protocols for use in different cell and tissue types.

## Methods

### Strains, media, and growth

All strains of *C. thermocellum* (LL345 and LL1111) and *T. saccharolyticum* (LL1025 and LL1076) used in this study were gifts of Lee Lynd (Dartmouth College) and his laboratory. Strain LL345 (Δ*hpt*) was used in all *C. thermocellum* metabolite extraction protocol validation experiments, unless otherwise listed. Strains of *C. thermocellum* were grown in MTC-5 media [5] and strains of *T. saccharolyticum* were grown in MTC-6 media [89]. Cultures were grown in 50 mL aliquots in 135-mL serum bottles containing a starting gaseous headspace of 5% H_2_, 10% CO_2_, and the balance N_2_. Cultures were grown to mid-log phase at 55 °C, shaking at 200 rpm. Cell growth was monitored by measuring OD_600_ measured in a Genesys 20 spectrophotometer (Thermo Fischer Scientific, Waltham, MA).

### Metabolite extractions

#### For determination of intracellular metabolites

**Fig. 5:**
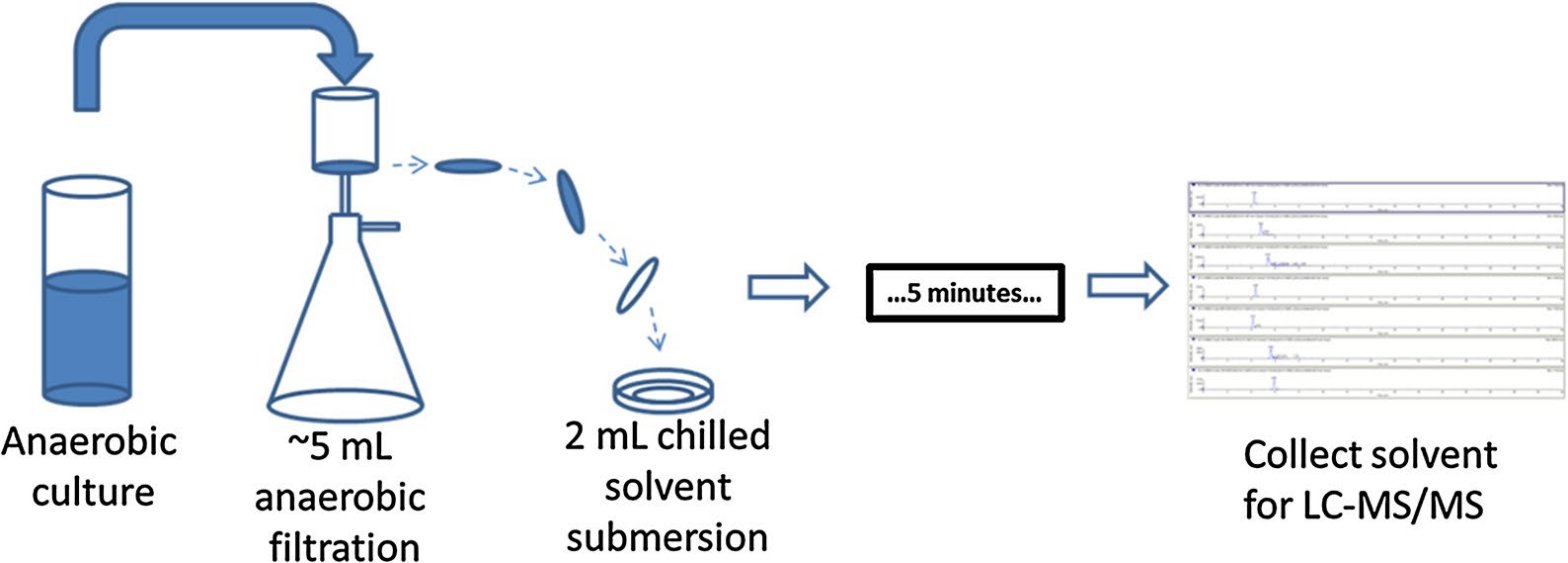
Diagram of the fast-filtering protocol used to extract and detect intracellular metabolites from cell biomass in this study

To determine intracellular metabolites, 5 mL of actively growing mid-log phase cells were quickly aspirated and vacuum filtered onto Whatman Nylon Membrane 0.22-µm Filters (GE Healthcare Life Sciences, 7404-004). The filters were then submerged (with the filter face containing cell biomass ‘down’) into 2 mL of extraction solvent (Fig. 5), consisting of 40% methanol (v)/40% acetonitrile (v)/20% water (v). The solvent was pre-chilled in a glass mini-petri dish (89000-300, VWR International, Radnor, PA) resting on top of an ice block which had been previously frozen at − 80 °C. The extraction solvent remained liquid throughout the extraction/submersion. Glass Pasteur pipettes (14672-380, VWR International, Radnor, PA) were used to collect extract and place extracts into pre-chilled silanized glass vials (MSCERT5000-S41 W, Thermo Fischer Scientific, Waltham, MA). Glass vials were pre-chilled by placing them in pellet ice for ~ 20 min prior to adding extract to them. The extract was kept on ice, in liquid form, and delivered the day of LC-MS/MS analysis.

Metabolomic sampling and extractions were done in a Coy anaerobic chamber (Type B, Coy Laboratory Products, Grass Lake, MI). All glassware was brought into the anaerobic chamber 24 h before metabolomic extractions to allow them to become anaerobic. Extraction solvent was prepared fresh for each extraction using HPLC grade solvents (water; WX0004-6, methanol; MX0488-6, acetonitrile; AX0142-6, VWR International, Radnor, PA). The solvent mixture was prepared, the headspace was sparged for 20 min with N_2_ gas, and it was stored overnight at − 20 °C in the dark. Extraction solvent was kept cold on pellet ice prior to use. Cell biomass from which metabolites were extracted (g CDW) was calculated using OD_600_ readings taken at the time of sampling and converted to cell dry weight using the conversion cited in [19].

#### For determination of metabolite losses due to handling

To determine metabolite losses due to handling, a mixture containing 1.66 µM of each metabolite was prepared and chilled in glass petri dishes as described previously. A fresh filter was adhered to the filter extraction apparatus and wetted with anaerobic water that had been treated using a Barnstead Nanopure Analytical Ultrapure Water System (D11901, Thermo Fisher Scientific, Waltham, MA). This filter was then placed into 2 mL of pre-chilled extraction solvent containing metabolites and allowed to incubate for 5 min, to simulate handling steps used, and approximate interferences from dilution, adsorption, degradation, etc., encountered during a typical extraction. Extraction solvent from the petri dish containing solvent and the wetted filter was collected and measured. Extraction solvent containing the spiked in metabolite, but had not been used in the ‘mock’ extraction, was also collected and metabolites quantified.

#### For determination of metabolite yield loss during storage at − 80 °C

Metabolite mixtures containing 1, 0.1, and 0.01 µM of each metabolite were prepared in extraction solvent and aliquoted into silanized glass vials. The vials were frozen at − 80 °C. At each prescribed sampling time, one of the aliquots was thawed and analyzed for metabolite concentrations. Vials were thawed and analyzed via LC–MS/MS at 0, 24, 48, and 120 h.

#### Extracting cell biomass multiple times to determine extraction efficacy

**Fig. 6:**
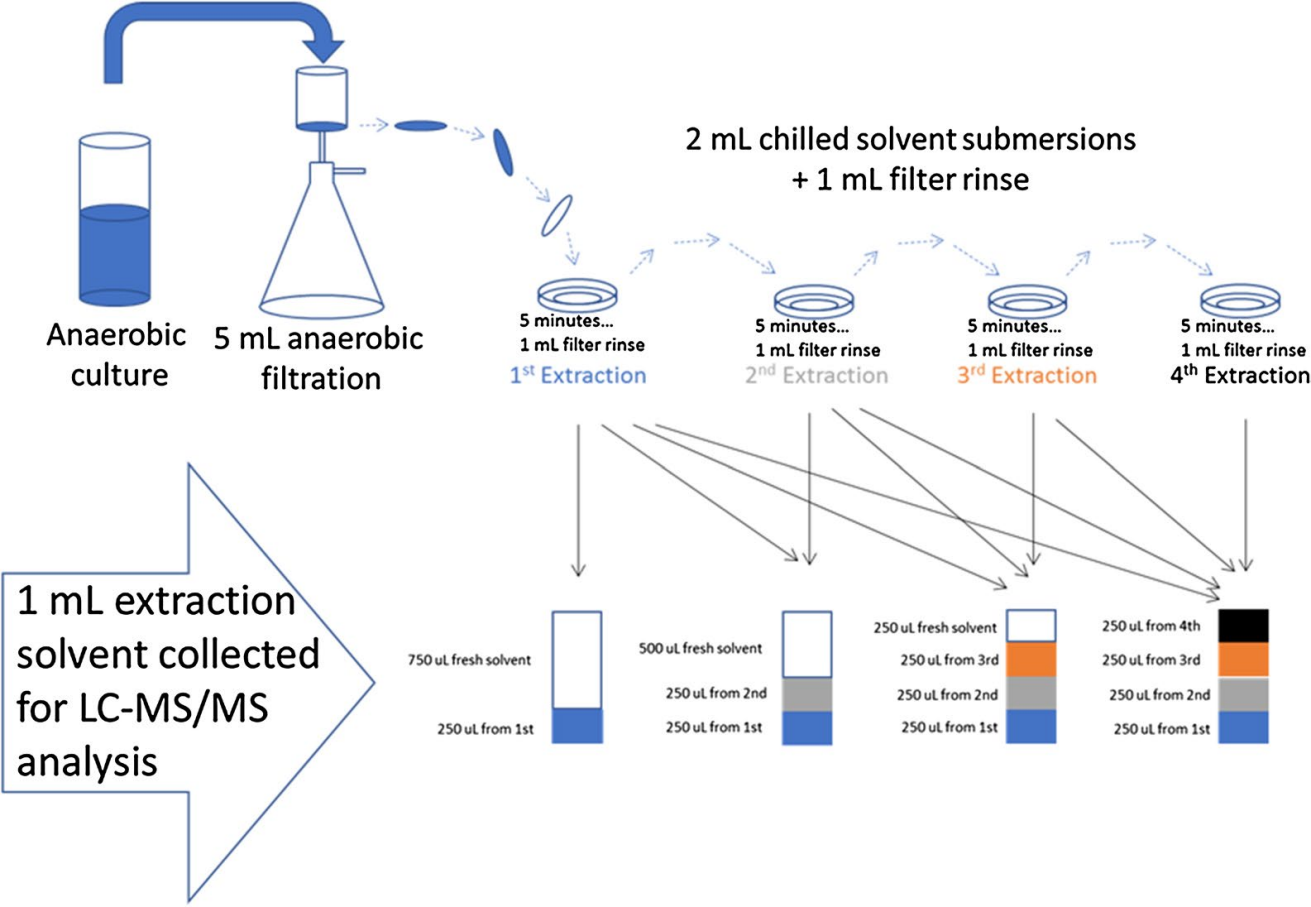
Diagram of method used to collect and aliquot cell extract from biomass that had been extracted multiple times

To determine metabolite extraction efficacy and examine whether multiple extractions would afford more complete/quantitative extraction, *C. thermocellum* cell biomass was extracted as described above. After incubating the filter containing cell biomass for 5 min in pre-chilled solvent, the filter was rinsed with an additional 1 mL of extraction solvent and placed into another glass petri dish containing 2 mL of fresh extraction solvent. The filter was rinsed and transferred two more times to fresh solvent, having the effect that the filter-laden cell biomass was exposed to fresh solvent four times sequentially. Samples were collected for LC–MS/MS analysis as shown in Fig. 6.

#### LC–MS/MS of intracellular metabolites

LC–MS/MS analyses were performed using a Waters Aquity UPLC system coupled to either an ABSciex 4000 QTrap or ABSciex 5500 QTrap mass spectrometer equipped with a TurboIon Spray source. The mass spectrometer was operated in negative ion mode using multiple reaction monitoring (MRM). Chromatographic separation of metabolites was attained on a 150 mm × 2.1 mm ID, 5 µm SeQuant ZIC® *p*HILIC column (part number 1.50460.0001, Merck from VWR) using acetonitrile (mobile phase A) and 10 mM ammonium carbonate in 0.2% (v/v) aqueous ammonium hydroxide (mobile phase B). Metabolite elution was performed using a gradient from 80% A to 60% B over 15 min and holding at 60% B for 5 min and then to 80% A for a 10-min equilibration period (30 min total run time) at a flow rate of 300 µL/min. Samples were diluted fivefold in 80/20 acetonitrile/water (v/v) and placed in an autosampler held at 4 °C. Sample volume injected onto the column was 5 µL.

The mass spectrometer settings were as follows: IonSpray voltage − 4.5 kV, curtain gas flow 20 (arb.), ion source gas 1 (nebulizer) flow 40 (arb.), ion source gas 2 (heating) flow 75 (arb.), nebulizing gas temperature 350 °C. Ionization and collision cell parameters were optimized separately for each metabolite and are shown in Table 3.

**Table 3:** Ionization and collision cell parameters used to analyze metabolites in this study

Metabolite	Product ion (*m/z*)	Declustering potential (DP) V	Collision energy (CE) eV	Cell exit potential (CXP) V
AMP	79	− 100	− 60	− 15
ADP	79	− 105	− 120	− 15
ATP	79	− 55	− 100	− 15
NAD	540.1	− 70	− 20	− 10
NADH	79	− 110	− 120	− 3
NADP	620.1	− 60	− 20	− 10
NADPH	79	− 110	− 115	− 5

#### Preparation of calibration curve

A concentrated stock solution (1 mM) of each metabolite standard was prepared in water. A concentrated mixture of metabolites (each 10 µM) was prepared by aliquoting the appropriate volume from each standard and diluting to a final volume of 5 mL in 80/20 acetonitrile/water (v/v). Serial dilutions were then made to obtain standard mixtures ranging from 0.01 to 1 µM. Five microlitre of each standard was injected onto the column. A linear calibration curve was generated by plotting the area response of the metabolite versus the concentration of the metabolite which was then used to determine the metabolite concentration in the cell extracts.

To determine yield loss of metabolites due to handling and storage, metabolite separation and analysis was done as described above, though analyzing either thawed or freshly prepared metabolite mixtures in place of a cell extract.

#### LC–MS/MS assessment of solvent and matrix-induced signal suppression of targeted metabolites

To assess for signal suppression from cell extract matrix, chromatographic and mass spectrophotometric instruments were used as described above, with modifications. A mixture containing 0.25 µM of each metabolite was prepared in fresh extraction solvent. This mixture was infused into the elution stream exiting the chromatography column (5 µL/min standard mixture via syringe pump to 300 µL/min HPLC mobile phase flowrate), generating a steady-state signal for each metabolite. Cell extract prepared, diluted, were injected and analyzed as stated above and signal suppression (indicated by deflections in the steady-state signal of each metabolite) was assessed at the predetermined retention time for each metabolite.
